# Assessing Recovery from Delirium: An International Survey of Healthcare Professionals Involved in Delirium Care

**DOI:** 10.56392/001c.56675

**Published:** 2022-12-19

**Authors:** Erin Noble, Haruno McCartney, Alasdair M MacLullich, Susan D Shenkin, Graciela Muniz-Terrera, Jonathan J Evans, Daniel Davis, Daisy Sandeman, Zoë Tieges

**Keywords:** delirium, delirium care, delirium recovery, repeat assessments, assessment tools, questionnaire, survey

## Abstract

**Background:**

A crucial part of delirium care is determining if the delirium episode has resolved. Yet, there is no clear evidence or consensus on which assessments clinicians should use to assess for delirium recovery.

**Objective:**

To evaluate current opinions from delirium specialists on assessment of delirium recovery.

**Design:**

Online questionnaire-based survey distributed internationally to healthcare professionals involved in delirium care.

**Methods:**

The survey covered methods for assessing recovery, the importance of different symptom domains for capturing recovery, and local guidance or pathways that recommend monitoring for delirium recovery.

**Results:**

Responses from 199 clinicians were collected. Respondents were from the UK (51%), US (13%), Australia (9%), Canada (7%), Ireland (7%) and 16 other countries. Most respondents were doctors (52%) and nurses (27%). Clinicians worked mostly in geriatrics (52%), ICUs (21%) and acute assessment units (17%). Ninety-four percent of respondents indicated that they conduct repeat delirium assessments (i.e., on ≥2 occasions) to monitor delirium recovery. The symptom domains considered most important for capturing recovery were: arousal (92%), inattention (84%), motor disturbance (84%), and hallucinations and delusions (83%). The most used tool for assessing recovery was the 4 ‘A’s Test (4AT, 51%), followed by the Confusion Assessment Method (CAM, 26%), the CAM for the ICU (CAM-ICU, 17%) and the Single Question in Delirium (SQiD, 11%). Twenty-eight percent used clinical features only. Less than half (45%) of clinicians reported having local guidance that recommends monitoring for delirium recovery.

**Conclusions:**

The survey results suggest a lack of standardisation regarding tools and methods used for repeat delirium assessment, despite consensus surrounding the key domains for capturing delirium recovery. These findings emphasise the need for further research to establish best practice for assessing delirium recovery.

## Introduction

Delirium is a severe neuropsychiatric syndrome in which there is acute deterioration in attention, level of arousal and other domains. Most cases of delirium last for 2-4 days, though up to 20% of cases may persist for weeks or longer.^1^

A crucial part of delirium care is determining if the delirium episode has resolved.^2^ This is essential to evaluate the effects of treatments, manage the risk of complications such as falls and inform discharge planning. It is good practice to inform patients and relatives of the diagnosis and the response to treatment.^3^ Outcomes of patients with persistent delirium, including institutionalisation and mortality, are worse.^1^

Delirium assessment tools are mostly designed to detect prevalent delirium on a single assessment, e.g., the 4 A’s Test (4AT)^4^ or Confusion Assessment Method (CAM),^5^ or for repeated monitoring of non-delirious patients for incident delirium, e.g., Recognising Acute Delirium As part of your Routine scale (RADAR),^6^ highlighting the lack of focus on delirium recovery. This may be due to the assumption that the natural course of delirium is transient, whereby delirium resolves over time without treatment. Subsequently, there is no clear evidence or recommendations as to which specific methods clinicians should use to assess for delirium recovery. This is compounded by the lack of consensus on the definition of delirium recovery, despite previous demonstration of need,^7^ which negatively impacts delirium care and characterisation of this syndrome.

Several uncertainties exist regarding best practice for repeat delirium assessments. Previous surveys within the field have focused either on clinicians’ general knowledge or opinions about delirium care.^8,9^ No survey to date has focused on delirium recovery. To address this knowledge gap, we surveyed current opinions from delirium specialists on assessment of delirium recovery.

## Methods

### Survey Development and Design

The questionnaire-based survey covered methods for assessing delirium, methods for assessing recovery, the importance of different symptom domains for capturing recovery, and local guidance or pathways recommending monitoring for recovery. Questions were developed, piloted and refined by the research team with clinical and/or research expertise in delirium, including nurse practitioners, geriatricians, statisticians and psychologists (see Supplementary Material 1 for the questionnaire).

### Survey Administration

This survey was aimed at healthcare practitioners and researchers interested in delirium care and research, including members of the European Delirium Association, the American Delirium Society and the Australasian Delirium Association. The survey was distributed online predominantly via Twitter and, to a lesser extent, via email. We collected responses from 16 March until 10 May 2021. All respondents provided informed consent for the potential use of their anonymised responses in publication. Completion time was approximately 5-10 minutes. Our study followed the Consensus-Based Checklist for Reporting of Survey Studies.^10^

### Statistical Analysis

We summarised variables using median and interquartile range (IQR) for continuous variables and percentages for categorical variables. The χ^2^ test was applied to compare the use of the most common methods (4AT, CAM and clinical features) for repeat assessments by profession (doctors compared to nurses), nationality (UK compared to non-UK respondents), and clinical experience (≤3 compared to >3 years of experience). All analyses were conducted using R (version 3.6.1, R Core Team, Vienna, Austria) and IBM SPSS Statistics (version 24.0, SPSS Inc., Chicago, USA).

## Results

A total of 206 responses were collected from a range of healthcare professionals from different healthcare settings. Seven respondents were excluded from analyses as they were not involved in clinical work (N = 4) or the clinical care of patients with delirium (N = 3), resulting in a final sample size of 199.

Respondents were from the UK (51%), US (13%), Australia (9%), Canada (7%), Ireland (7%) and 16 other countries. Most were doctors (52%) and nurses (27%) working mainly in geriatrics (52%), Intensive Care Units (21%) and assessment units (17%). The median number of years of active clinical work since obtaining a primary professional qualification was 15 (IQR = 8-20) (65% with >10 years of clinical experience). Table 1 shows respondent demographics by country, profession and clinical setting; full information is provided in Supplementary Material 2.

For delirium assessment, 95% of respondents reported using specific methods. Practitioners mostly used the 4AT (67%), the CAM (29%), the Single Question to Identify Delirium (SQiD) (25%) and the CAM-ICU (21%); multiple response options could be selected. It was also common for delirium to be assessed via clinical features only (without using a named scale) (22%) and via the Diagnostic Statistical Manual, 5^th^ Edition (DSM-5) criteria for delirium (11%). Twenty-three other tools/methods each accounted for ≤7% of responses.

Regarding recovery, 177 of 189 (94%) respondents indicated that they sometimes assess patients on ≥2 occasions to monitor whether the delirium had improved or worsened (Table 2). For these repeat assessments, the most used tests were the 4AT (51%), the CAM (26%), the CAM-ICU (17%) and the SQiD (11%). However, repeat assessments were also common to involve only clinical features (28%). Other methods each accounted for ≤9% of responses (Table 2).

Overall, 143 of 178 (80%) respondents reported using the same tool(s) or method(s) for the first and second delirium assessment; 9% reported changing to abbreviated tools or a subset of items; 7% reported using different tools or methods; and 4% reported other approaches being used, such as “clinical judgement”, “assessing change over time” and “comparing presentation [of symptoms] to previous input”.

UK clinicians were significantly more likely than non-UK clinicians to use the 4AT (UK: 81%, non-UK: 45%, *p*<0.001) and non-UK clinicians were more likely to use the CAM (UK: 9%, non-UK: 46%, *p*<0.001) for repeat assessments of delirium. UK clinicians were more likely than non-UK clinicians to use clinical features alone (UK: 27%, non-UK: 15%, *p*=0.048).

Senior clinicians (>3 years of clinical experience) were more likely than junior clinicians (≤3 years of clinical experience) to use the 4AT (senior: 66%, junior: 38%, *p* =0.011). There was no significant relationship between clinical experience and use of CAM or clinical features.

Doctors were more likely than nurses to use clinical features to assess for delirium recovery (doctors: 33%, nurses: 8%, *p*<0.001) but there was no relationship between profession and use of either the 4AT or the CAM. Other professions were excluded from these analyses as they comprised a low percentage of the respondent pool (21% combined).

**Figure 1:**
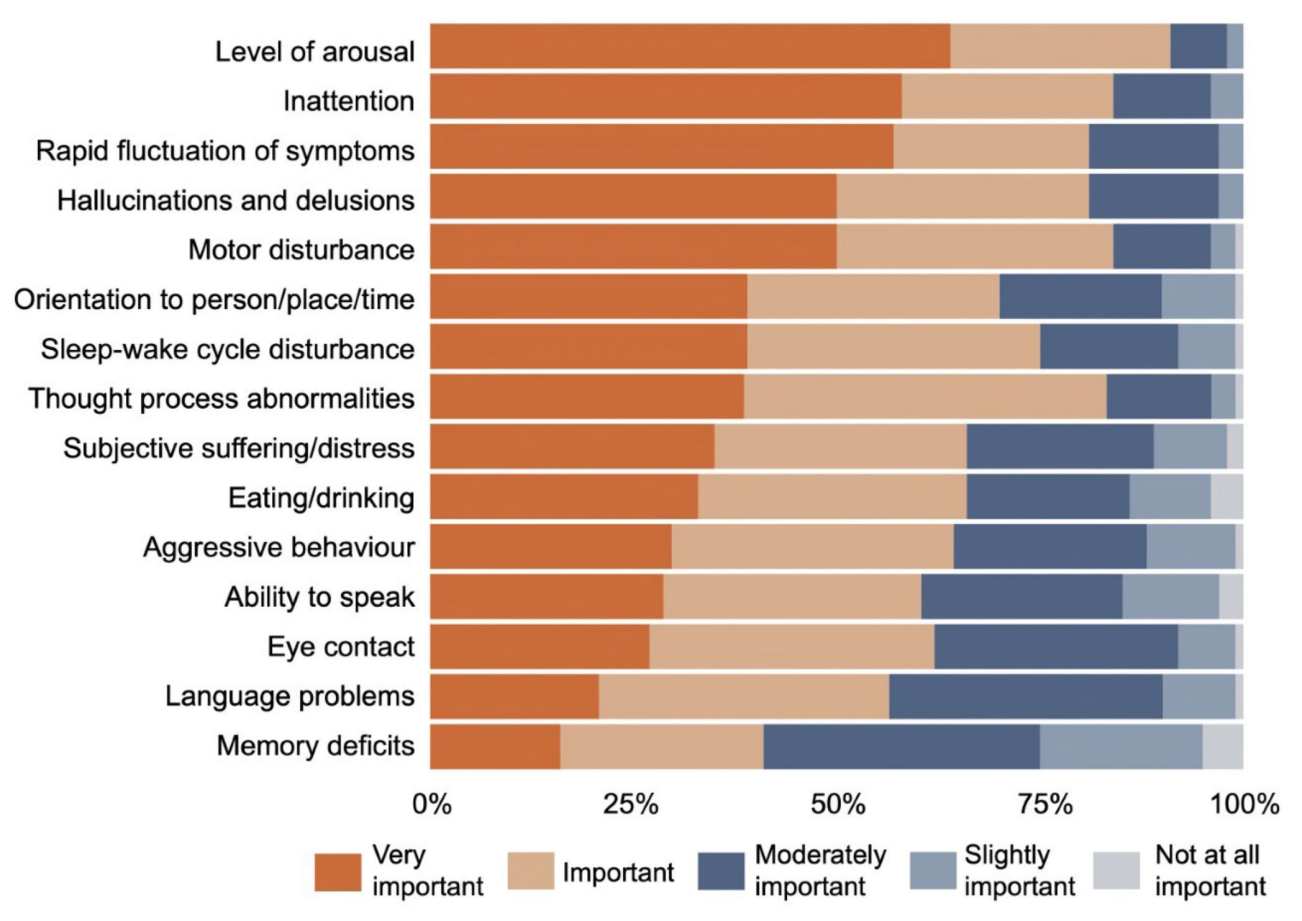
Importance of symptom domains in capturing recovery from delirium, as indicated by respondents.

There was consensus on which symptom domains show changes that best reflect delirium recovery: arousal (92% agreement i.e., respondents rating the symptom domain as important or very important), inattention (84%), motor disturbance (84%), thought process abnormalities (84%), hallucinations and delusions (82%) and rapid fluctuations of symptoms (81%). Memory deficit was considered the least important symptom domain (41%) (Figure 1).

Responses were mixed as to whether clinicians had local guidance or pathways within their professional units which recommend monitoring for delirium recovery; 45% answered yes, 44% answered no and 11% did not know. Within the guidance in use, 74% of respondents indicated that specific methods were recommended for assessing delirium recovery, primarily the 4AT (43%), the CAM-ICU (21%) and the CAM (18%). Eighteen percent of respondents indicated that no specific tools/methods were recommended in their local guidance and 8% did not know.

Barriers to assessing recovery from delirium were lack of time, not seeing patients again due to working patterns and not having enough information to judge (Table 3). Several respondents highlighted that the lack of time had worsened during the COVID-19 pandemic, e.g.: “[there is] now much less time to do daily reassessments”.

Clinicians agreed that there is currently a lack of stan-dardisation in assessing delirium recovery. Responses included: “there is no standard approach”, “a lack of consensus of how many delirium assessments or for how long they should be negative to define delirium recovery”, and “a lack of awareness of how to assess and help patients recover from delirium”. Respondents also highlighted that recovery is “not prioritised before discharge”, and patients are “still delirious at discharge”. When asked how they judge patients’ recovery from delirium, clinicians indicated that improvement in specific symptom domains is important (“less agitation”, “more alert”, etc.).

## Discussion

This survey suggests considerable variability in how clinicians approach assessment for recovery of delirium and the extent to which guidance on this issue is in place. Ninety-five percent of clinicians used specific tools or methods to assess for delirium, with the 4AT being the most common, followed by the CAM, SQiD and CAM-ICU. A similar proportion (94%) of clinicians indicated that they sometimes assess patients for delirium on ≥2 occasions to monitor if the delirious episode improves or worsens. For repeat assessments, the 4AT was the most used tool. Less than half of clinicians reported having local guidance or pathways in their unit which recommend monitoring for delirium recovery.

While 80% of respondents reported using the same tool(s) or method(s) for the first and second delirium assessment, others relied on abbreviated tools, different tools, subsets of items, or no tools at all (i.e., clinical judgement alone). The tools and methods used at repeat assessment also differed by type of respondent: UK clinicians were more likely than non-UK clinicians to use the 4AT and less likely to use the CAM, senior clinicians were more likely than junior clinicians to use the 4AT, and doctors were more likely than nurses to use clinical features.

There was consensus among clinicians that arousal, inattention, motor disturbance, thought process abnormalities, hallucinations and delusions, and rapid fluctuation of symptoms are the most important domains for capturing delirium recovery; memory was considered the least important (though still considered important by 41% of respondents). Clinicians’ judgement of delirium recovery focused mainly on improvement of these specific symptom domains; there was no consistent indication of methods being used to inform clinicians’ judgement of delirium recovery. Clinicians also indicated that lack of time, not seeing patients again due to working patterns and lack of information to make a judgement were common barriers to assessing delirium recovery.

These findings align with previous work in the field, which has demonstrated variability among definitions of delirium recovery.^7^

Despite some limitations, this is the largest online survey exploring clinicians’ attitudes and approaches towards delirium recovery. Using a sample specifically of clinicians interested in delirium will have likely resulted in selection bias. Moreover, as we conducted the survey online, the denominator is unknown; hence, the response rate could not be reliably ascertained. There is also the subjectivity of responses; current outcomes only reflect this group of clinicians’ views on measuring delirium recovery. However, the diversity of professions and settings from which clinicians were recruited offers a broad range of perspectives on delirium recovery.

Many respondents were UK-based and international differences in nomenclature may impact responses. The USA may be more inclined to use ‘acute encephalopathy’ instead of ‘delirium’.^11^ However, those who indicated that they were not specifically involved in ‘delirium care’ were excluded (implying respondents recognise the terminology ‘delirium’ to be in line with other definitions such as ‘acute encephalopathy’). Issues surrounding delirium terminology may extend to clinicians’ approaches to assessing recovery, reflective of the overarching heterogeneity of the condition of delirium in general.

Despite our efforts to avoid biased responses, it is possible that the wording of the questions and response options may have impacted responses. Question phrasing was considered to ensure that respondents were not primed to mirror the wording of the question within their answer, e.g., ‘recovering’ not ‘improving’ was used. We used preset phrases to gauge the most common barriers to assessing delirium recovery. What clinicians understood these phrases to represent is unclear, again highlighting the issue of subjectivity. Some may interpret the phrase ‘lack of time’ as a proxy term for ‘not important’, i.e., they do not priori-tise assessing delirium recovery, as opposed to not having the time to carry out repeat assessments.

These findings have implications for clinical practice and future research. The survey showed a lack of consensus amongst clinicians on what constitutes ‘recovery’ from delirium. Many viewed delirium recovery similarly to diagnosis, i.e., binary, whereby patients are classified as delirium-positive or delirium-negative.^12^ Yet, categorising patients in this way is not always possible.^13^ The binary approach may also be less applicable to delirium recovery due to the fluctuating nature of delirium (one delirium-negative result may not accurately reflect recovery). Cole et al^14^ suggested that delirium recovery could be considered as a continuum, with patients deemed fully, partially or not recovered based on the presence of none, several or all delirium symptoms. Scales allowing grading of delirium features may have more utility in assessing recovery than binary scales. Such scales may ultimately contribute to better characterisation of the delirium syndrome and an increased awareness of the graded nature of recovery.

A significant issue in measuring delirium recovery is that it is unclear how changes in assessment scores over time (e.g., hours versus days versus weeks) should be interpreted as ‘recovery’ from delirium in clinical practice. Current tools (e.g., the 4AT) can be administered repeatedly to patients on separate occasions, but there is no explicit guidance on how changes in test scores can be operationalised to reflect a patient’s improvement or decline, and what constitutes a clinically meaningful change. Establishing the tools and methods that can be operationalised for repeat use by clinical staff to measure delirium recovery would assist in both clinical practice and research.

The survey results inform further research on delirium recovery. Specifically, validating standardised assessment tools and methods for delirium recovery in clinical settings should be actively pursued. The most common barrier to assessing delirium recovery was lack of time; hence any validated tool must be effective at quickly and comprehensively evaluating recovery in clinical practice. Given that the 4AT was the most utilised tool for both delirium assessment and repeat assessment in this survey, there may be scope for the 4AT to be explored as a potential instrument for assessing delirium recovery in clinical settings.

This survey highlighted clinicians’ opinions on assessing delirium recovery, using a sample of healthcare professionals involved in delirium care. Clinicians recognised the importance of repeated assessment in tracking delirium recovery and showed agreement on the key domains for capturing delirium recovery. Despite this, implementation of repeat delirium assessments was highly variable. A lack of formal consensus or evidence-based guidance regarding tests most appropriate for repeat delirium assessments remains a key challenge. Standardisation of approach to repeat assessment of delirium and consistent documentation in the clinical record should be pursued to establish best practice.

## Supplementary Material

Supplementary Material 1

Supplementary Material 2

Supplementary Material 3

## Figures and Tables

**Table 1:** Respondent demographics to online survey of delirium recovery (N = 199)

		N respondents	%
**Country**	UK	101	50.8
	US	26	13.1
	Australia	17	8.5
	Canada	14	7.0
	Ireland	13	6.5
	Other	28	14.1
			
**Profession**	Doctor	103	51.8
	Nurse	53	26.6
	Occupational therapist	18	9.0
	Other (e.g., Physiotherapist, Advanced paramedic; Dietitian; Critical care physician assistant; Critical care rehabilitation practitioner)	25	12.6
			
**Setting**	Geriatric medicine	103	51.8
	Critical care	41	20.6
	Acute assessment/medical assessment unit	34	17.1
	Rehabilitation ward	31	15.6
	Surgical ward (not including orthopaedics)	28	14.1
	Internal medicine specialist ward (e.g., Cardiology, Respiratory)	21	10.6
	Liaison mental health	21	10.6
	Emergency department	20	10.1
	Orthopaedics	18	9.0
	Stroke	18	9.0
	Other (e.g., Primary care; Inpatient psychiatry ward; Paediatric critical care; Community mental health team)	18	9.0
	Hospice/palliative care	8	4.0
	Oncology	5	2.5
	Old age mental health ward	4	2.0

**Table 2:** Tools and methods used for first and repeat delirium assessments (multiple response question) (N=199)

	First assessment		Repeat assessment	
Tool/method	N	%	N	%
4AT	127	66.8	90*	51.1*
Confusion Assessment Method (CAM)	55	28.9	45*	25.6*
Single Question in Delirium (SQiD)	48	25.3	20	11.4
Clinical features only	42	22.1	50*	28.4*
CAM-ICU	40	21.1	29	16.5
DSM diagnostic criteria	20	10.5	10	5.7
Other	14	7.4	16	9.1
Ultra-Brief CAM (UB-CAM)	11	5.8	8	4.5
3D-CAM	8	4.2	9	5.1
Nursing Delirium Screening Scale (NU-DESC)	7	3.7	5	2.8
Delirium Rating Scale-Revised 98 (DRS-R98)	6	3.2	4	2.3
Memorial Delirium Assessment Scale (MDAS)	6	3.2	6	3.4
Delirium Observation Screening Scale (DOSS)	5	2.6	2	1.1
Delirium Index	2	1.1	3	1.7
Recognising Acute Delirium As part of your Routine (RADAR)	1	0.5	N/A	N/A

* The three most used tools for assessing delirium recovery.

**Table 3:** Clinicians’ most common barriers to assessing recovery from delirium in patients (N=199)

	Lack of time		My work pattern means that I don't see patients again		I don't have enough information to judge	
	N	%	N	%	N	%
Not at all common	39	19.5	72	36	85	42.5
Slightly common	65	32.5	57	28.5	73	36.5
Common	58	29	38	19	25	12.5
Fairly common	21	10.5	17	8.5	13	6.5
Very common	17	8.5	16	8	4	2

## References

[1] Whitby J, Nitchingham A, Caplan G, Davis D, Tsui A (2022). Persistent delirium in older hospital patients: an updated systematic review and meta-analysis. Delirium.

[2] Scottish Intercollegiate Guidelines Network (2022). Risk Reduction and Management of Delirium.

[3] The National Institute for Health and Care Excellence (2022). Delirium: Prevention, Diagnosis and Management.

[4] Shenkin SD, Fox C, Godfrey M (2019). Delirium Detection in Older Acute Medical Inpatients: A Multicentre Prospective Comparative Diagnostic Test Accuracy Study of the 4AT and the Confusion Assessment Method. BMC Med.

[5] Inouye SK, van Dyck CH, Alessi CA, Balkin S, Siegal AP, Horwitz RI (1990). Clarifying Confusion: The Confusion Assessment Method. A New Method for Detection of Delirium. Ann Intern Med.

[6] Voyer P, Champoux N, Desrosiers J (2015). Recognizing Acute Delirium as Part of your Routine [RADAR]: A Validation Study. BMC Nurs.

[7] Adamis D, Devaney A, Shanahan E, McCarthy G, Meagher D (2014). Defining ‘Recovery’ for Delirium Research: A Systematic Review. Age and Ageing.

[8] Jenkin RPL, Al-Attar A, Richardson S, Myint PK, MacLullich AMJ, Davis DHJ (2016). Increasing Delirium Skills at the Front Door: Results from a Repeated Survey on Delirium Knowledge and Attitudes. Age and Ageing.

[9] Morandi A, Davis D, Taylor JK (2013). Consensus and Variations in Opinions on Delirium Care: A Survey of European Delirium Specialists. Int Psychogeriatr.

[10] Sharma A, Minh Duc NT, Luu Lam Thang T (2021). A Consensus-Based Checklist for Reporting of Survey Studies (CROSS). Journal of General Internal Medicine.

[11] Slooter AJC, Otte WM, Devlin JW (2020). Updated Nomenclature of Delirium and Acute Encephalopathy: Statement of Ten Societies. Intensive Care Med.

[12] Ely EW, Inouye SK, Bernard GR (2001). Delirium in Mechanically Ventilated Patients Validity and Reliability of the Confusion Assessment Method for the Intensive Care Unit (CAM-ICU). JAMA.

[13] Larsen LK, Frøkjaer VG, Nielsen JS (2018). Delirium assessment in neuro-critically ill patients: A validation study. Acta Anaesthesiol Scand.

[14] Cole MG, McCusker J, Bailey R (2016). Partial and No Recovery from Delirium After Hospital Discharge Predict Increased Adverse Events. Age and Ageing.

