# Supplementary material for: Assessing Recovery from Delirium: An International Survey of Healthcare Professionals Involved in Delirium Care
Source: Delirium Commun. Author manuscript; Available in PMC 2023 Mar 25. (PMC7614362; doi:10.56392/001c.56675)
Supplement: Supplementary Material 1 [file EMS172112-supplement-Supplementary_Material_1.pdf]

Assessing recovery from delirium – a survey of healthcare practitioners.

Introduction

Who are the intended respondents?

This survey is intended for healthcare practitioners with an active interest in delirium care and research, including (but not limited to) members of the European Delirium Association (EDA), the American Delirium Society (ADS) and the Australasian Delirium Association (ADA).

Please do not fill in this survey if you are not a healthcare practitioner with an interest in delirium.

Why this survey?

A crucial part of delirium care is determining if the delirium episode has resolved. This is essential to allow clinicians to evaluate the effects of treatments, to manage the risk of complications such as falls and dehydration, and to inform discharge planning. It is also a mandatory part of good practice to inform patients and relatives of the diagnosis and the response to treatment.

Yet there is no clear evidence on what tests clinical staff should use to assess for delirium recovery.

As part of our efforts to improve delirium assessment and treatment, we are interested in finding out more about opinions on assessment of delirium recovery.

What does the survey cover?

Attitudes and opinions of assessing recovery from delirium.

Which institutions are involved?

Team members are employed by the University of Edinburgh, the University of Glasgow,

and University College London in the UK.

Are my responses anonymous?

The survey is completely anonymised. No computer location information will be collected. There are some broad questions on your professional background, the type of setting you work in, and the country in which you work. The results of this survey will be published and some anonymised responses may be quoted in these publications.

How long will the survey take?

Less than 5 minutes.

Introduction

If you are interested in helping with the survey, please read the first question below and if appropriate, click 'I agree'.

1. Do you agree to take part in this survey and consent to the potential use of your anonymised responses as described above? ✱ *Required*

- I agree

General information

What is your current profession? (primarily, but tick more than one option if your time is divided). ✱ *Required*

- [ ] Doctor
- [ ] Nurse
- [ ] Occupational therapist
- [ ] Physiotherapist
- [ ] Other

If you selected Other, please specify:

Which clinical setting(s) do you currently work in? (primarily, but tick more than one option if your time is divided). * ✱ *Required*

Please select between 1 and 14 answers.

- [ ] Emergency department
- [ ] Acute assessment/ medical assessment unit
- [ ] Critical care
- [ ] Geriatric medicine
- [ ] Orthopaedics
- [ ] Surgical ward (not including orthopaedics)
- [ ] Internal medicine specialist ward (e.g. cardiology, respiratory, gastroenterology, nephrology, endocrinology, neurology)
- [ ] Oncology
- [ ] Stroke

- [ ] Rehabilitation ward
- [ ] Hospice/ palliative care
- [ ] Old age mental health ward
- [ ] Liaison mental health
- [ ] Not involved in clinical work
- [ ] Other (please specify)

If you selected Other, please specify:

General information

How many years have you been working (approximate full-time equivalent) since obtaining your primary professional qualification? ✱ *Required*

Please enter a whole number (integer).

Please make sure the number is between 0 and 100.

In what country do you currently work? ✱ *Required*

Are you involved in the clinical care of patients with delirium? ✱ *Required*

- Yes
- No

Methods for assessing delirium

Do you use tools or methods to assess for delirium? ✱ *Required*

- Yes
- No

Methods for assessing delirium

Which tool(s) or method(s) do you routinely use to assess for delirium in your clinical practice? Please tick all that apply. ✱ *Required*

- ☐ Single Question to identify Delirium (SQiD)
- ☐ 4AT
- ☐ Confusion Assessment Method (CAM)
- ☐ 3D-CAM
- ☐ Ultra-Brief CAM (UB-CAM)
- ☐ CAM-ICU
- ☐ RADAR
- ☐ Nursing Delirium Screening Scale (NU-DESC)
- ☐ Delirium Observation Screening Scale (DOSS)
- ☐ NEECHAM Confusion Scale
- ☐ Memorial Delirium Assessment Scale (MDAS)
- ☐ Delirium Rating Scale-Revised 98 (DRS-R98)
- ☐ Delirium Index
- ☐ DSM diagnostic criteria
- ☐ Clinical features only without using a named scale
- ☐ Other

If you selected Other, please specify:

Methods of assessing recovery from delirium

The following questions concern **assessment of recovery from delirium in clinical practice**.

Please answer according to your own views. These questions are not intended to test knowledge but rather to understand more about existing variations in clinical and academic opinion and practice.

Do you sometimes assess a patient for delirium on two or more occasions, with the aim to monitor if their delirium is improving or getting worse (i.e. repeating delirium assessments in the same patient over time)? ✱ *Required*

- Yes
- No
- Other

If you selected Other, please specify:

Methods of assessing recovery from delirium

Which tool(s) or method(s) do you use for repeat assessments of delirium in clinical practice?

- ☐ Single Question to identify Delirium (SQiD)
- ☐ 4AT
- ☐ Confusion Assessment Method (CAM)
- ☐ 3D-CAM
- ☐ Ultra-Brief CAM (UB-CAM)
- ☐ CAM-ICU
- ☐ RADAR
- ☐ Nursing Delirium Screening Scale (NU-DESC)
- ☐ Delirium Observation Screening Scale (DOSS)
- ☐ NEECHAM Confusion Scale
- ☐ Memorial Delirium Assessment Scale (MDAS)
- ☐ Delirium Rating Scale-Revised 98 (DRS-R98)
- ☐ Delirium Index
- ☐ DSM diagnostic criteria
- ☐ Clinical features only without using a named scale
- ☐ Other

If you selected Other, please specify:

Do you use the same or different tool(s) or method(s) for the first assessment and for the second assessment(s)? ✱ *Required*

- ○ Same tool(s)/method(s) for first and second assessment
- ○ Different tool(s)/method(s) for first and second assessment
- ○ Abbreviated tool(s)/method(s) for the second assessment, or a subset of tool items
- ○ Other

If you selected Other, please specify:

Methods of assessing recovery from delirium

In your practice, how common are the following barriers to assessing recovery from delirium in patients? ✱ *Required*

Please don't select more than 1 answer(s) per row.

Please select at least 3 answer(s).

	Not at all common	Slightly common	Common	Fairly common	Very common
Lack of time	☐	☐	☐	☐	☐
My work pattern means that I don't see patients again	☐	☐	☐	☐	☐
I don’t have enough information to judge	☐	☐	☐	☐	☐

In your experience, how can you tell if someone is recovering from delirium?

Please indicate the importance of the following symptom domains in capturing recovery from delirium. ✱ *Required*

Please don't select more than 1 answer(s) per row.

Please select at least 15 answer(s).

	Not at all important	Slightly important	Moderately important	Important	Very important

Ability to speak	☐	☐	☐	☐	☐
Eye contact	☐	☐	☐	☐	☐
Eating/drinking	☐	☐	☐	☐	☐
Level of arousal (e.g. drowsy)	☐	☐	☐	☐	☐
Sleep-wake cycle disturbance	☐	☐	☐	☐	☐
Motor disturbance (agitation or retardation)	☐	☐	☐	☐	☐
Hallucinations and delusions (including paranoia)	☐	☐	☐	☐	☐
Orientation to person, place or time	☐	☐	☐	☐	☐
Memory deficits	☐	☐	☐	☐	☐
Aggressive behaviour	☐	☐	☐	☐	☐
Inattention	☐	☐	☐	☐	☐
Subjective suffering or distress	☐	☐	☐	☐	☐
Thought process abnormalities (disconnected thought, rambling etc.)	☐	☐	☐	☐	☐
Language problems (fluency, word-finding, slurred speech etc.)	☐	☐	☐	☐	☐
Rapid fluctuation of symptoms	☐	☐	☐	☐	☐

Guidance and frameworks

Do you have local guidance/frameworks on delirium in your unit which recommend monitoring for recovery from delirium?

- Yes
- No
- Don't know

Guidance and frameworks

Do the local guidance/frameworks recommend specific tools or methods for assessing recovery from delirium?

- Yes
- No
- Don't know

Guidance and frameworks

Which tools or methods are recommended in the guidance/frameworks for assessing recovery from delirium?

Comments

Please let us know of any issues surrounding delirium recovery and its assessment that have not been addressed.

End of survey

Your responses have been submitted.

Thank you for your time and effort!
