# Supplementary material for: Assessing Recovery from Delirium: An International Survey of Healthcare Professionals Involved in Delirium Care
Source: Delirium Commun. Author manuscript; Available in PMC 2023 Mar 25. (PMC7614362; doi:10.56392/001c.56675)
Supplement: Supplementary Material 2 [file EMS172112-supplement-Supplementary_Material_2.pdf]

Supplementary Material 2. Full table of respondent demographics to online survey of delirium recovery (N = 199).

		N respondents	%
**Country**	UK	101	50.8
	US	26	13.1
	Australia	17	8.5
	Canada	14	7.0
	Ireland	13	6.5
	Italy	5	2.5
	Mexico	4	2.0
	Spain	3	1.5
	France	2	1.0
	Germany	2	1.0
	Pakistan	2	1.0
	Argentina	1	0.5
	Brazil	1	0.5
	Chile	1	0.5
	Dominican Republic	1	0.5
	India	1	0.5
	Japan	1	0.5
	Lebanon	1	0.5
	The Netherlands	1	0.5
	New Zealand	1	0.5
	Turkey	1	0.5
**Profession**	Doctor	103	51.8
	Nurse	53	26.6
	Occupational therapist	18	9.0
	Other (e.g., Advanced paramedic; Dietitian; Critical care physician assistant; Critical care rehabilitation practitioner)	15	7.5
	Physiotherapist	10	5.0
**Setting**	Geriatric medicine	103	51.8
	Critical care	41	20.6
	Acute assessment/medical assessment unit	34	17.1
	Rehabilitation ward	31	15.6
	Surgical ward (not including orthopaedics)	28	14.1
	Internal medicine specialist ward (e.g., Cardiology, Respiratory)	21	10.6
	Liaison mental health	21	10.6
	Emergency department	20	10.1
	Orthopaedics	18	9.0
	Stroke	18	9.0
	Other (e.g., Primary care; Inpatient psychiatry ward; Paediatric critical care; Community mental health team)	18	9.0
	Hospice/palliative care	8	4.0

	Oncology	5	2.5
	Old age mental health ward	4	2.0
